# Direct Conversion of Human Dermal Fibroblasts into Cardiomyocyte‐Like Cells Using CiCMC Nanogels Coupled with Cardiac Transcription Factors and a Nucleoside Drug

**DOI:** 10.1002/advs.201901818

**Published:** 2020-02-07

**Authors:** Hye Jin Kim, Hyun Jyung Oh, Ji Sun Park, Jung Sun Lee, Jae‐Hwan Kim, Keun‐Hong Park

**Affiliations:** ^1^ Nano‐Regenerative Medical Engineering Department of Biomedical Science College of Life Science CHA University 618, CHA Biocomplex, Sampyeong‐Dong, Bundang‐gu Seongnam‐si 13488 Republic of Korea; ^2^ Molecular Genetics Department of Biomedical Science College of Life Science CHA University 605, CHA Biocomplex, Sampyeong‐Dong, Bundang‐gu Seongnam‐si 13488 Republic of Korea

**Keywords:** 5‐azacytidine, carboxymethyl cellulose, cardiogenic‐specific factors, induced cardiomyocytes, nanogels

## Abstract

Using direct conversion technology, normal adult somatic cells can be routinely switched from their original cell type into specific differentiated cell types by inducing the expression of differentiation‐related transcription factors. In this study, normal human dermal fibroblasts (NHDFs) are directly converted into cardiomyocyte‐like cells by drug and gene delivery using carboxymethylcellulose (CMC) nanoparticles (CiCMC‐NPs). CMC‐based multifunctional nanogels containing specific cardiomyocyte‐related genes are designed and fabricated, including *GATA4*, *MEF2C*, and *TBX5* (GMT). However, GMT alone is insufficient, at least in vitro, in human fibroblasts. Hence, to inhibit proliferation and to induce differentiation, 5‐azacytidine (5‐AZA) is conjugated to the hydroxyl group of CMC in CiCMC‐NPs containing GMT; in addition, the CMC is coated with polyethylenimine. It is confirmed that the CiCMC‐NPs have nanogel properties, and that they exhibit the characteristic effects of 5‐AZA and GMT. When CiCMC‐NPs‐containing 5‐AZA and GMT are introduced into NHDFs, cardiomyocyte differentiation is initiated. In the reprogrammed cells, the mature cardiac‐specific markers cardiac troponin I and α‐actinin are expressed at twofold to threefold higher levels than in NHDFs. Engineered cells transplanted into live hearts exhibit active pumping ability within 1 day. Histology and immunohistology of heart tissue confirm the presence of transplanted engineered NHDF cells at injection sites.

## Graphical abstract

Cardiomyocyte‐inducing carboxymethylcellulose nanoparticles are biocompatible nanogel‐type nanoparticles used as a dual delivery system in normal human dermal fibroblasts.

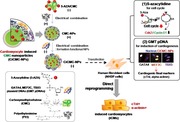

## 1 Introduction

Heart failure, a major health problem associated with critical complications, is one of the most lethal diseases in the world.^1^ When cardiomyocyte cells are damaged, they cannot be regenerated to restore the beating action of the heart. Therefore, complete heart transplantation is the only way to cure heart failure. However, this procedure is technically challenging, limited by the small number of available donors, and has severe side effects associated with the accompanying immunosuppressive therapy. To overcome these issues, cell therapy has been proposed as an alternative to transplantation. However, this approach does not yet meet the functional criteria for current clinical trials.^2^ Therefore, research efforts have been devoted to differentiating somatic cells into functional cardiomyocyte cells by tissue engineering, followed by grafting of the engineered cells.^3^

The direct conversion method can switch normal human dermal fibroblasts (NHDFs) to another mature cell type. Many research groups have based their efforts on a novel concept of differentiation, distinct from the induced pluripotent stem cells (iPSCs) method using the four factors identified by Yamanaka. Instead, these scientists have directly redifferentiated cells without taking them through an iPSC stage.^4^ In this study, we directly reprogrammed NHDFs into cardiomyocytes using mature cells. In direct reprogramming, transcription factors and drugs are the mediators of differentiation. Therefore, suitable particles are needed to carry these agents into the target cell.^5^

Nano‐sized materials have been investigated as carriers for simple and safe delivery of genes and drugs into cells. Due to their small size, these materials can easily penetrate cells without any obstructions.^6^ Nanocarriers must be biocompatible with cells, tissues, and the human body as a whole. In addition, they must be safe, high in water content, easy to manufacture, and they must also have a high protein‐loading efficiency.^7^ A material that satisfies all of these properties is carboxymethylcellulose (CMC), an environmentally friendly and non‐toxic saccharide. CMC is water‐soluble and has a temperature specificity, making it suitable for use as a carrier for drugs and proteins.^8^ When carboxymethyl cellulose (CMC) is dissolved in water, the hydroxyl group of the glucose residues in cellulose can be substituted, making it possible to conjugate CMC to various types of drugs with cationic groups.^9^ Similarly, the carboxyl groups in CMC can interact with the cationic groups of amines. In previous studies of gene delivery, our group formed complexes of a typical cationic polymer, polyethylenimine (PEI), to the carboxyl groups of CMC.^9^ The “polyplexed” PEI/CMC easily complexes with genes and drugs, and can be easily transferred into human mesenchymal stem cells via endocytosis.^10^

Transcription factors are directly used for the differentiation process as the research on the factors expressing in the process of growing from the embryonic state to the adult cell became active.^11^ The factors that differentiate fibroblasts into cardiomyocytes include MyoD, which promotes differentiation into muscle.^12^ Recently, the transcription factors GATA4, MEF2C, and TBX5 (collectively, GMT) were used to directly differentiate mouse fibroblasts into myocardial cells.^13^ Initially, many studies have focused on GMT conduction alone. Induced cardiomyocytes (iCMs) are produced less efficiently by GMT, but upon addition of Hand2, the efficiency increases, and both GMT and GMTH decrease remodeling following myocardial infarction and cardiac dysfunction.^14^ The efficiency of reprogramming can be further increased by adding Mesp1, Hand1, Hand2, Nkx2.5, and Myocard (Myocd).^15^ In this study, we differentiated NHDFs into iCMs using GMT, the main factors involved in inducing differentiation into cardiomyocytes.^16^ In addition, due to the low efficiency of differentiation using transcription factors alone, we combined these factors with 5‐azacytidine (5‐AZA) to further promote cardiomyocyte production.

5‐AZA, an inhibitor of proliferation, demethylates DNA, thereby weakening the effects of gene‐silencing mechanisms mediated by methylation.^17^ Demethylation decreases the stability of the silencing signal, resulting in gene activation. Thus, 5‐AZA is used not only to study tumor degeneration, but also to promote the differentiation of adult stem cells into cardiomyocytes.^18^ In combination with GMT, 5‐AZA is injected intracellularly to induce cross‐differentiation directly into cardiomyocytes with greater efficiency.^19^

We developed delivery vehicles capable of self‐assembly with DNA through formation of a complex with the cationic group of 5‐AZA and electrostatic interactions with strong cationic polymers. When these vehicles are used to introduce transcription factors and drug NHDFs, they activate differentiation into cardiomyocytes. Therefore, we transfected cardiomyocyte induced CMC‐nanoparticles (CiCMC‐NPs) into human fibroblasts and monitored the cells for evidence of direct conversion.

**Scheme 1:**
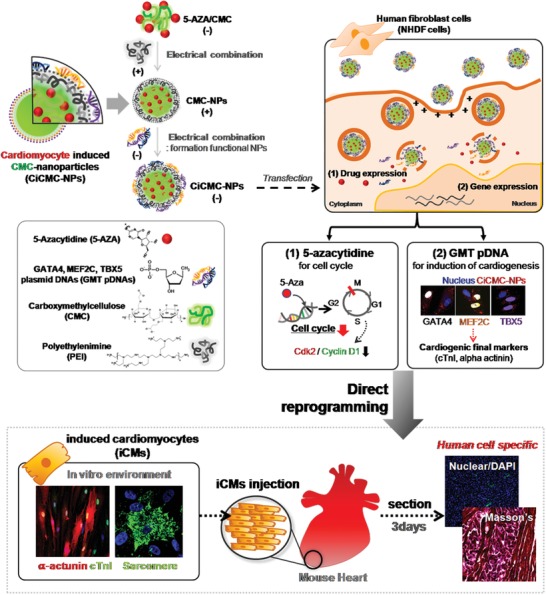
Schematic of direct reprogramming of normal human dermal fibroblasts (NHDFs) using nanogel‐type CMC complexed with DNA encoding three cardiogenic factors (GATA4, MEF2C, and TBX5) and a chemical drug (5‐azacytidine) to promote DNA demethylation and cell arrest.

In the process of cardiomyocyte differentiation using CiCMC‐NPs, cardiac‐like phenotypes are induced in human fibroblasts (**Scheme** **1**). For example, iCMs express multiple cardiac markers and exhibit spontaneous contractility for some period of time. According to various studies, the cardiac markers GMT are expressed when cells are differentiated from embryonic cardiomyocytes, and GMT also promotes expression of NKx2.5.^20^ In the late stage, cardiac troponin I (cTnI) and alpha‐actinin are expressed.

## 2 Results and Discussion

### 2.1 Synthesis of CiCMC‐NPs with the Temperature and pH Properties of Nanogels

**Figure 1:**
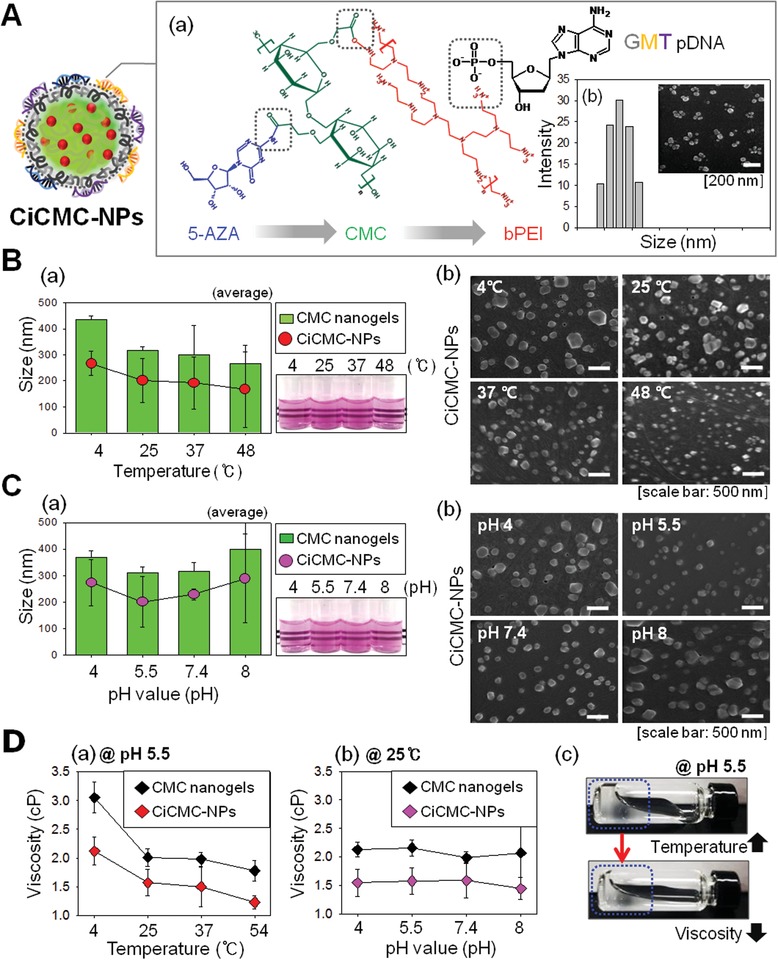
Characteristics of CiCMC‐NPs in terms of the properties of CMC. A) Schematic of CiCMC‐NPs. a) Detailed diagram of CiCMC‐NPs. b) Sizes of CiCMC‐NPs. B) Sizes and morphologies of CiCMC‐NPs as a function of temperature. a) Size, as determined by DLS and turbidity. b) Morphology, as determined by SEM. C) Sizes and morphologies of CiCMC‐NPs as a function of pH. a) Size, as determined by DLS and turbidity. b) Morphology, as determined by SEM. D) Viscosities of CiCMC‐NPs as a function of a) temperature and b) pH; c) images of the change in nanogel viscosity.

We fabricated novel nano‐sized gel‐type nanoparticles for use in direct conversion of somatic cells into myocardial cells. The newly generated nanoparticles were simultaneously loaded with a drug (5‐AZA) and complexed with genes (GMT plasmid DNA [pDNA]) that can induce differentiation into cardiomyocytes. The combination of CMC nanogels and both of these agents is important for efficient differentiation into cardiomyocyte cells, because particle size changes depending on CMC nanogel concentration and the CMC–polymer bond ratio. We confirmed that, when 0.1% CMC nanogel and polymer were bonded at a ratio of 1:1, they formed nano‐sized gels (Figure S1, Supporting Information). New nanoparticles were formed by ionic bonding. All of the materials used in this approach were water‐soluble and had individual charges. 5‐AZA ionically bonded to the carboxyl groups of CMC nanogels via amine groups. Because GMT pDNA has a negative charge, it cannot complex directly with CMC nanogels. Therefore, we first complexed the nanogels with a cationic polymer, PEI, which has amine groups; this caused the nanogels to carry a positive charge, enabling electrostatic interactions with GMT pDNA. This interaction was confirmed by the sizes and morphologies of CiCMC‐NPs, which can be verified in detail in **Figure** **1**A. All bonds in the CiCMC‐NPs were ionic bonds (Figure 1A,a). We confirmed that the CiCMC‐NPs were of nanometer scale through dynamic light scattering (DLS) and scanning electron microscopy (SEM) (Figure 1A,b). Monitoring of size and morphology at each step during the production of CiCMC‐NPs indicated that the polymer had nanoscale dimensions starting from the time of complex formation (Figure S2, Supporting Information). When the amine groups of 5‐AZA bind to the ester groups of CMC by electrostatic attraction, the CMC is reduced in size to about 320 nm because the hydrophobic moieties are toward to the core of nanogels. In addition, when PEI with another strong amine group is complexed with residue ester groups of CMC that are not bound with 5‐AZA, it forms a nanogel about 190 nm smaller than that of CMC complexed with 5‐AZA due to its core entanglement. However, if the core‐concentrated nanogels form complexes with pDNAs, the size of the nanogels increases up to 266 nm. This means that the entangled cation groups and anion groups in the core portion were loosened and the loosened cation groups were headed outwards while they were bound to the phosphate groups of the pDNA.

Figure 1B–D shows that CiCMC‐NPs were of nanometer scale, but the exact size changed depending on temperature and pH. Our CiCMC‐NPs were fabricated based on CMC, which is itself sensitive to both of these physical factors.^21^ Therefore, we sought to determine whether CiCMC‐NPs were similar to CMC in terms of their pH and temperature dependence. To this end, we investigated how their sizes changed with temperatures and pH. CMC nanogels decreased in size as temperature increased, and CiCMC‐NPs exhibited similar trends. According to DLS measurements, CMC nanogels had dimensions of 400, 300, 300, and 250 nm at 4, 25, 37, and 48 °C, respectively, and CiCMC‐NPs had dimensions of 280, 230, 220, and 190 nm, respectively (Figure 1B,a). Thus, CiCMC‐NPs were of optimal size for endocytosis. The morphology‐ and temperature‐dependent trends were confirmed in greater detail by SEM (Figure 1B,b). Both CMC‐NPs and CiCMC‐NPs were sensitive to temperature and had sizes that were suitable for application to cells as the temperature increased. Other properties of nanogels include sensitivity to pH. Size changed less dramatically in response to pH than to temperature (Figure 1C,a). CMC nanogels were 350, 300, 310, and 400 nm at pH of 4, 5.5, 7.4, and 8, respectively, and CiCMC‐NPs were 290, 210, 250, and 300 nm, respectively. These results show that the particles were significantly smaller at pH 5.5, which is the pH in the cytoplasm, indicating that CiCMC‐NPs are suitable nanoparticles for in‐cell applications. In addition, we confirmed that the temperature is similar to that of CMC nanogels. Morphological analysis confirmed the size change (Figure 1C,b).

Another common feature of nanogels is their viscosity. Hence, we investigated how viscosity changed in response to temperature and pH. In both CMC nanogels and CiCMC‐NPs, the viscosity of the nanogel decreased with increasing temperature, but was not affected by pH (Figure 1D,a,b). The reduction in viscosity at higher temperatures could be observed visually. As the temperature increased, gelation decreased and the gel sank (Figure 1D,c).

### 2.2 Identification of CiCMC‐NPs with 5‐AZA in the Cell Cycle

**Figure 2:**
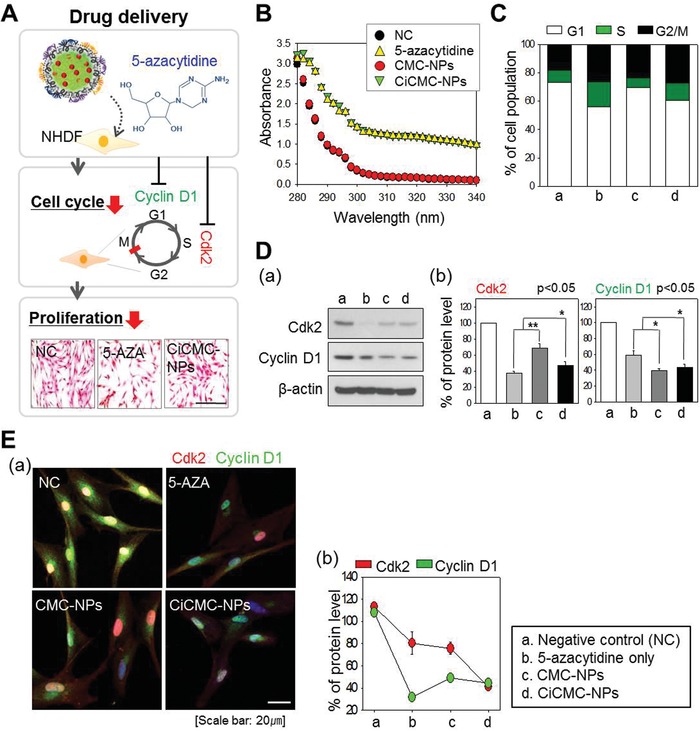
Characterization of 5‐AZA‐complexed CMC‐NPs. A) Schematic of the effect of 5‐AZA, including cell cycle arrest and downregulation of cell cycle proteins (cyclin D1 and Cdk2). After drug treatment, cell proliferation ceased. B,C) Cell cycle arrest. FACS revealed a high proportion of cells in G2. D) Western blots and expression levels of cell cycle proteins. E) Immunofluorescence staining. a) Confocal microscopy. b) Graph of expression levels.

Next, we tried to induce differentiation of NHDFs into cardiomyocytes by transfecting the cells with CiCMC‐NPs containing 5‐AZA and GMT pDNA, both of which promote differentiation into cardiomyocytes. 5‐AZA inhibits DNA methyltransferase of stem cells caused to cease the proliferation and differentiation at low concentrations. However, at high concentrations, it is known to cause cytotoxicity and cell death. Therefore, the useful concentration of 5‐AZA in the case of cardiomyogenesis has been reported to be about 10–20 µM. Based on this study, we prepared and analyzed CiCMC nanogels with 5‐AZA at various concentrations (10–200 µM). Since 10–20 µM 5‐AZA, which is a useful concentration in cardiomyocyte differentiation, forms nanoparticles of less than 200 nm, it is a suitable nano‐sized material for use as a delivery vehicle for drugs and genes to cells (Figure S2, Supporting Information). However, for CiCMC nanogel formations at different concentrations of 50, 100, and 200 µM of 5‐AZA and CMC, the results of the DLS analysis showed that the nanoparticles were widely formed over ranges from 400 to 650 nm (Figure S3, Supporting Information). Nanogels formed to a size of 200 nm or more are thought to be less effective than CMC‐nanogels formed by 10–20 µM of 5‐AZA as drug‐delivery vehicles. As a result of cell cycle arrest analysis using the CiCMC nanogels into cells, the fraction of G2/M phase formed by 20 µM 5‐AZA‐coupled nanogel was about 19.2%, which is about a 10% increase compared to the control group. However, the 10 µM 5‐AZA‐coupled CMC‐nanogel showed an increase of about 5% over the 14.5% fraction on G2/M. Thus, DLS and cell cycle arrest assay results show that the 20 µM 5‐AZA‐coupled CMC‐nanogels are optimal for nanoparticle size and potency of 5‐AZA (Figure S3, Supporting Information). When the CiCMC‐NPs were transfected into NHDF cells, 5‐AZA caused cell cycle arrest (**Figure** **2**A). When cell cycle progression is blocked, levels of Cdk2, which regulates S phase, and cyclin D1, which regulates G1 phase, are reduced. Hence, the cells ceased proliferating, as confirmed by hematoxylin and eosin (H&E) staining.

We then investigated in greater detail the effects of 5‐AZA in the cell. The presence of 5‐AZA in CiCMC‐NPs was determined by UV spectroscopy (Figure 2B). At a wavelength of 270 nm, negative control (NC) and GMT pDNA with CMC‐NPs decreased the sharpness of the peak and 5‐AZA was presented. The sample group had weak absorbance, confirming that the absorbance properties varied depending on the presence of 5‐AZA. Thus, the absorbance of CiCMC‐NPs was similar to that of 5‐AZA alone, indicating that 5‐AZA was present in the particles.

Next, we experimentally confirmed the effect of 5‐AZA in CiCMC‐NPs on the cell cycle (Figure 2C–E). First, we confirmed cell cycle arrest using FACS. Compared with the NC group, cells treated with 5‐AZA‐containing CiCMC‐NPs (Figure 2C,b,d) increased the proportion of cells in G2/M stage (black) and G1 stage (white) (Figure 2C). When cell cycle arrest occurred, the levels of factors regulating the cell cycle also changed. Changes in Cdk2 and Cyclin D1, associated with G1/S phase, were observed at the protein level. Western blot analysis revealed that the intensities of the Cdk2 and Cyclin D1 bands (Figure 2D,b,d) were reduced in the presence of 5‐AZA relative to the NC group (Figure 2D,a). In a parallel experiment, protein level was confirmed using a confocal microscope: Cdk2 is shown in red, and Cyclin D1 in green; both fluorescence intensities were weaker than in NC (Figure 2E,a). Quantitation of fluorescence intensity revealed that cyclin D1 levels were lowest in 5‐AZA‐treated cells, and that Cdk2 levels were lowest in cells treated with CiCMC‐NPs (Figure 2E,b).

Therefore, 5‐AZA was efficiently loaded into the nanogels, and when the particles were introduced into cells, they affected the cell cycle similarly to treatment with the drug alone. Thus, we confirmed that the cellular effects of 5‐AZA were preserved in CiCMC‐NPs.

### 2.3 Cell Uptake of Human GATA4, TBX5, and MEF2C Expression Vectors

**Figure 3:**
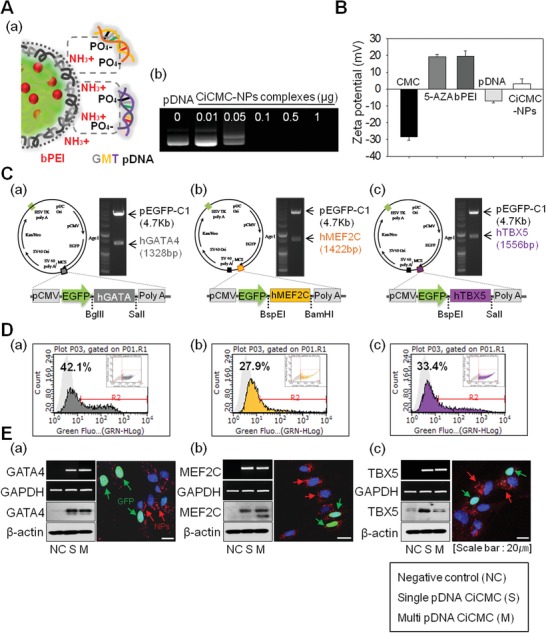
Characterization of cardiogenic vectors (GATA4, MEF2C, and TBX5) in complex with CiCMC‐NPs by RT‐PCR, Western blot, FACS, and confocal microscopy. A) Zeta‐potential due to the formation of CiCMC‐NPs. B,a) Scheme of interaction between cationic CMC‐NPs and anionic GMT pDNA. C) Maps of expression vectors generated by recombinant PCR methods. D) FACS analysis of the efficiency of pEGFP‐encoding vectors. E) Transfection efficiency of NHDFs using CiCMC‐NPs, at the mRNA and protein levels, as determined by confocal microscopy. Vector: a) GATA4, b) MEF2C, and c) TBX5.

Next, we tested CiCMC‐NPs complexed with both 5‐AZA and plasmids that induce cardiomyocyte differentiation (**Figure** **3**). GMT pDNAs were synthesized by cloning into key vectors, and then used to generate CiCMC‐NPs. Figure 3a illustrates how GMT pDNA vectors were complexed with CiCMC‐NPs: specifically, GMT pDNA vectors were complexed with CMC‐NPs (CMC nanogels mediated with PEI). Due to the carboxyl groups in CMC, it was necessary to use materials with amine groups in order to achieve complexation with both nanogel and pDNA vectors. Because PEI has appropriate amine groups, the PEI/CMC nanogels complexed with anionic pDNA vectors, yielding complete CiCMC‐NPs (Figure 3A,a). Gel retardation analysis revealed that the optimal vector concentration for CMC‐NP binding to pDNA vectors was 0.1 µg. Hence, to confirm that CiCMC‐NPs were produced, GMT pDNA vectors were conjugated to CMC‐NPs at a concentration of ≥0.1 µg (Figure 3A,b). When we measured several types of samples, we could confirm that PEI/CMC nanogels complexed with pDNA had positive charges, whereas pDNAs and CMC had negative charges (Figure 3B).

Next, we cloned GMT pDNA vectors and fabricated complexes of these DNAs with CiCMC‐NPs. The expression vectors used in this study were generated by recombinant PCR methods. Briefly, human *GATA4* and *TBX5* cDNA clones were purchased from Dharmacon (Dharmacon, Lafayette, CO, USA), and a *MEF2C* clone was purchased from Korea Human Gene Bank (https://genbank.kribb.re.kr, Korea Research Institute of Bioscience & Biotechnology, South Korea). The cDNA clones were amplified by PCR, and then ligated into the multiple cloning site (MCS) of pEGFP‐C1 followed by restriction digestion (*Bgl*II/*Sal*I for *GATA4*, *Bsp*EI/*Sal*I for *TBX5*, and *Bsp*EI/*Bam*H1 for *MEF2C*). All constructs were verified by sequence analysis (Figure 3C,a–c).

Next, we tested the efficiency of introduction of GMT pDNA into NHDFs by CiCMC‐NPs (Figure 3D,E). Because each vector also encoded EGFP, the efficiency of gene transfer could be confirmed by monitoring fluorescence by FACS or confocal microscopy. Based on these measurements, efficiency was 42% for *GATA4*, 27% for *MEF2C*, and 33% for *TBX5* (Figure 3D,a–c). The vectors were localized to the nucleus, and the CiCMC‐NPs (which had red fluorescence) were distributed around the nucleus (Figure S4, Supporting Information). In parallel, we confirmed expression of each gene at the mRNA and protein level, and found that vector efficiency did not differ significantly when CiCMC‐NPs were in complex with one vector (single, Sin) versus both vectors (multiple, Mul). Hence, the vectors were transfected into NHDFs using CiCMC‐NPs. The degree of expression from each vector was similar (Figure 3E,a–c). When pDNA was introduced into cells using CiCMC‐NPs, the GMT vectors efficiently induced cardiomyocyte differentiation. Therefore, we conclude that CiCMC‐NPs are appropriate for use as nanoparticles to induce differentiation of fibroblasts into cardiomyocytes.

### 2.4 Confirmation of Direct Conversion through Cardiogenic Markers

**Figure 4:**
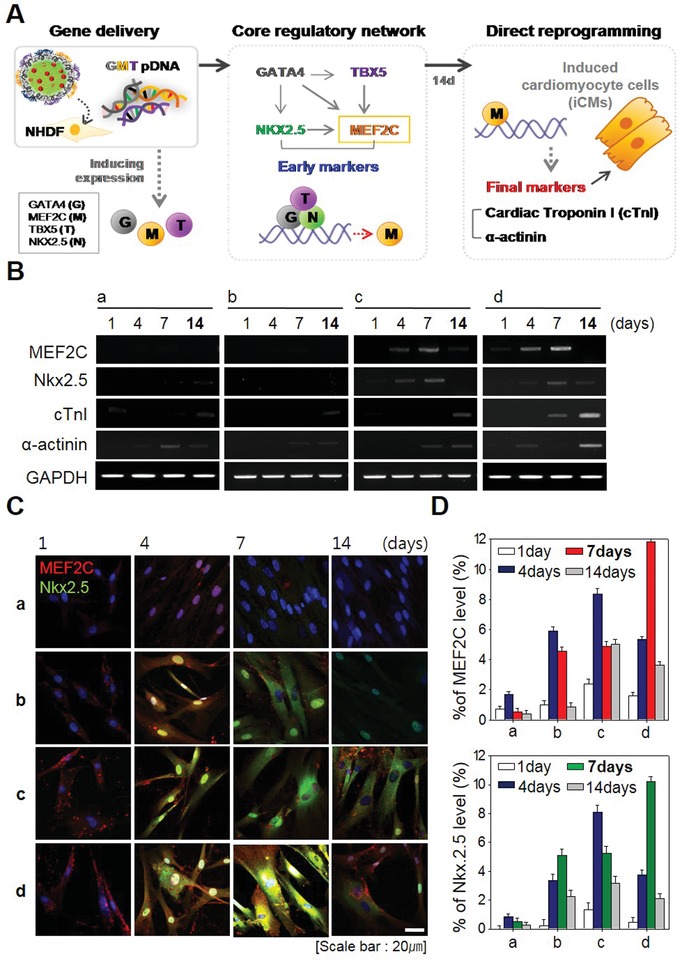
Expression of cardiac‐specific genes after treatment with CiCMC‐NPs. A) Summary of the molecular mechanism of cardiomyocyte proliferation. B) RT‐PCR of cardiac‐specific genes, comparing early markers with late markers as a function of time. C) Immunofluorescence staining of early markers by time. D) Protein levels of MEF2C and Nkx2.5 (%) about C: a) NC; b) CMC with 5‐AZA; c) CMC with GMT; and d) CiCMC.

In this study, we fabricated CiCMC‐NPs to generate a system capable of transferring genes and drugs that promote direct conversion of NHDF cells. In the experiments described above, we investigated the function and efficiency of these drugs and genes in nanoparticles. The nanoparticles were as efficient as either drugs or genes alone, indicating that they were suitable for induction of cardiomyocyte differentiation. **Figure** **4**A shows a simplified representation of differentiation markers. When CiCMC‐NPs were transferred to NHDF cells, GMT pDNA was expressed in the cells. *Nkx2.5* and *MEF2C* were expressed under the control of *GATA4*, as part of the core network. Expression of *MEF2C*, *Nkx2.5*, and *TBX5* induces differentiation into cardiomyocytes, in which various markers are expressed. *Nkx2.5* and *MEF2C*, early markers of heart development, were expressed at early times after induction. When NHDFs transfected with GMT were differentiated into induced cardiac cells (iCMs) 14 days after transfection, troponin I and α‐actinin were expressed. Over two different time periods, we confirmed that GMT‐transfected NHDF cells were differentiated into CiCMC‐NPs.

Figure 4B shows mRNA expression of cardiac markers when iCM differentiation was induced by CiCMC‐NPs as well as PEI/CMC nanogels with 5‐AZA or PEI/CMC nanogels with GMT alone. Cardiac marker mRNAs were expressed in transfected NHDF cells when differentiation was induced with either CiCMC‐NPs or with 5‐AZA or GMT (Figure 4B). CiCMC‐NPs induced the differentiation of NHDFs cells into iCMs more easily than CMCs loaded with 5‐AZA or coated with GMT pDNA. Cardiac markers were expressed at relatively high levels in both early and late stages of cardiomyocyte development. RT‐PCR analysis revealed that early markers of cardiomyocytes were strongly expressed on day 7, indicating that CiCMC‐NPs drove expression more strongly than cells induced with 5‐AZA or GMT alone. Because *MEF2C* was introduced via NPs, it was also slightly expressed on day 1. Expression of *MEF2C* was further amplified by expression of *Nkx2.5*. Following induction, the late markers were strongly expressed on day 14, at a time point when expression of the early markers *MEF2C* and *Nkx2.5* had begun to fade away. In addition, CiCMC‐NPs drove higher levels of expression than 5‐AZA or GMT alone. This result indicates that CiCMC‐NPs are powerful tools for induction of differentiation.

In addition, we monitored protein levels by immunofluorescence. The protein levels of early markers MEF2C and Nkx2.5 mirrored the RT‐PCR results: Nkx2.5 (green) and MEF2C (red) were highly expressed in cells transfected with CiCMC‐NPs (Figure 4C). CMCs containing only one factor, such as 5‐AZA or GMT pDNA alone, yielded only weak expression, and ultimately did not reach the levels achieved by CiCMC‐NPs containing both factors. Thus, once again, the transfer of two factors was superior to the transfer of a single factor. Quantitative analysis of early expression markers as a function of time yielded the same results (Figure 4D). Following transfection with CiCMC‐NPs, expression of MEF2C and Nkx2.5 was significantly elevated on day 7. By day 14, however, MEF2C and Nkx2.5 were almost undetectable, whereas the protein levels of late markers mirrored their mRNA levels (Figure S5, Supporting Information). Expression of early factors was higher with the dual delivery system than when only one factor was introduced, indicating that the late markers expressed when differentiated into myocardial cells were expressed compared to the positive control (PC).

### 2.5 Expression of Late Cardiac Markers as a PC

**Figure 5:**
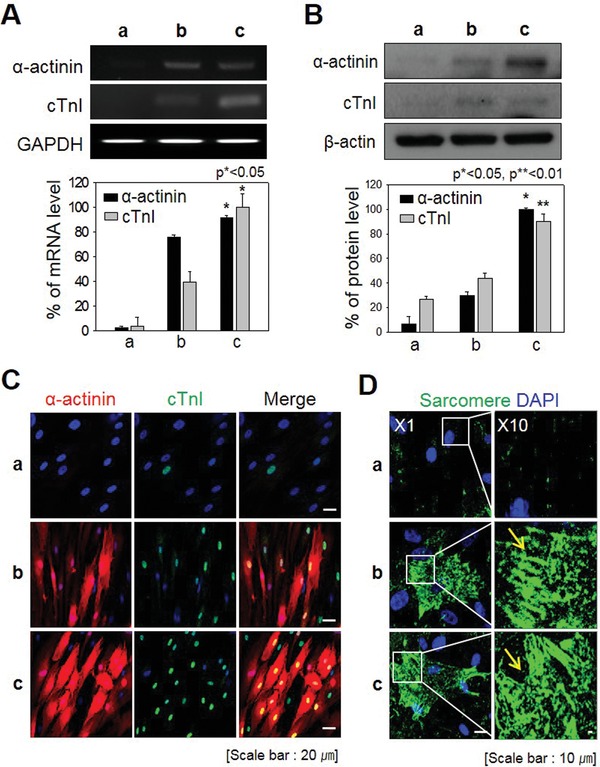
Expression of late cardiac markers relative to positive controls. A) mRNA levels of α‐actinin and cTnI (*p* < 0.05). B) Protein levels of α‐actinin and cTnI (*p* < 0.05 and *p* < 0.01). C) Immunofluorescence staining for α‐actinin and cTnI. D) Sarcomere staining to confirm myocyte morphology. a) NC; b) CiCMC; and c) PC.

CiCMC‐NP‐induced iCMs expressed cardiogenic markers with the greatest efficiency among all sample groups examined. Early markers were strongly expressed on day 7, but declined by day 14, when late markers were expressed. Hence, we compared the experimental and benign models 14 days after induction of iCM differentiation (**Figure** **5**). The CiCMC‐NPs drove higher levels of marker genes than the NC, and the expression levels were similar to those in the PC. At the mRNA level, α‐actinin and cTnI were expressed more strongly in the PC than in the NC (Figure 5A). These findings were mirrored at the protein level (Figure 5B).

Immunofluorescence revealed that late markers of cardiomyocytes were expressed at similar levels in the experimental group and PC (Figure 5C). As in previous experiments, the morphologies and marker expression patterns differed between the PC and NC. α‐Actinin (red) formed characteristic thread‐like fibers in the cytoplasm, and cTnI (green) was strongly localized to the nucleus.

Cardiomyocyte cells are the muscle cells that make up the heart, and thus contain long chains of specialized organelles called sarcomeres. In this sense, cardiomyocyte cells have streak patterns similar to those of skeletal muscle cells and can be identified by immunofluorescence. To further confirm that iCMs were similar to the PC, we compared the sarcomere pattern of iCMs and PC by sarcomere dyeing. At a magnification of ×10, it was clear that the cells contained stripes, indicating that they were muscle cells containing sarcomeres (Figure 5D).

### 2.6 Evaluation of iCMs in Mouse Hearts Using Human‐Specific Staining

**Figure 6:**
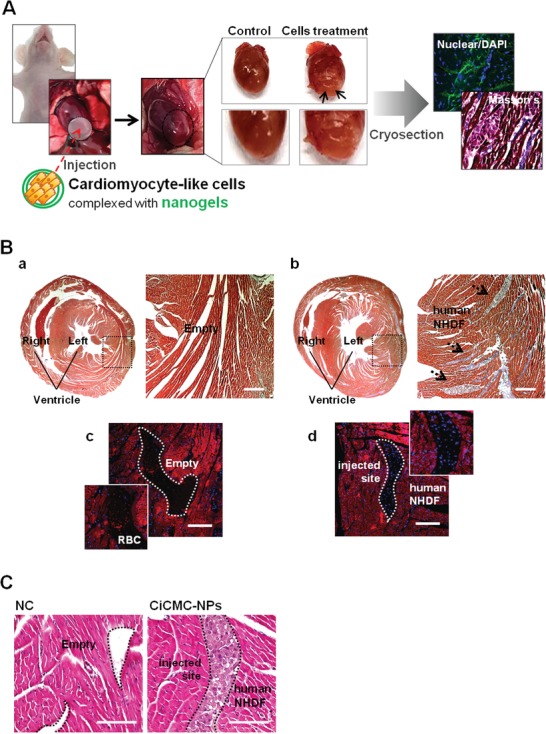
Ablation after injection of cardiomyocyte‐like cells engineered with CiCMC. A) Full view of the heart after CiCMC injection, Masson trichrome staining (MTS) of sectioned slides, and fluorescence staining. B) MTS and DAPI staining: a,c): NC and b,d) CiCMC. C) H&E staining: a): NC and b): CiCMC.

To evaluate cardiac muscle function, we tested the effects of NHDFs transfected with genes and drug after isolation of mouse hearts and intramyocardial injection of NHDFs (**Figure** **6**A). CiCMC‐NPs harboring GMT and 5‐AZA significantly increased cardiac beating relative to NHDFs treated with particles not containing either factor (Video S1, Supporting Information). The cardiac beating occurred as early as 1 h after injection, consistent with ex vivo monitoring, which revealed beating activity for 1 day.

In addition, we conducted histological analyses to verify the presence of implanted NHDFs at injection sites, and to monitor the presence of muscle in hearts (Figure 6B). Consistent with the video observations, NHDFs transfected with genes and drug exhibited high levels of collagen deposition; this production of extracellular matrix (ECM) in the heart indicated that the transplanted cells had survived (Figure 6B,b), in contrast to NHDFs treated without GMT genes and 5‐AZA (Figure 6B,a). Also, the presence of transplanted cells in the heart significantly increased the deposition of collagens near the transplant sites. The transplanted cells, observed by 4′,6‐diamidino‐2‐phenylindole (DAPI) staining (Figure 6B,d), were clearly present at injection sites.

To further verify the presence of exogenous cells, we stained the transplanted cells with H&E and observed them by confocal microscopy (Figure 6C). The images confirmed that exogenous cells were present in isolated mouse hearts (Figure 6C,b). They were compactly settled, whereas vacant spaces were observed in control hearts (Figure 6C,a, b).

## 3 Conclusion

In summary, we used nanogels complexed with drug (5‐AZA) and pDNA (GMT) to directly convert NHDFs into cardiomyocyte‐like cells. Our novel nanoparticle material could effectively deliver both pDNA and drugs into cells, resulting in successful cardiomyocyte differentiation. We anticipate that this approach will be useful in regenerative medicine.

## 4 Experimental Section

##### Material

CMC (MW = 90 000 and substitution degree = 0.8–0.9), 5‐AZA, and branched poly(ethyleneimine) (bPEI, MW = 25 000) were obtained from Sigma (Steinheim, Germany). pDNA‐encoding enhanced green fluorescent reporter protein (pEGFP) was obtained from Clontech. Dulbecco's modified Eagle's medium, high glucose (DMEM‐high), fetal bovine serum (10%), and Dulbecco's phosphate‐buffered saline (DPBS, pH 7.4) were purchased from Hyclone. Antibiotics and trypsin–EDTA (0.05%) were obtained from Invitrogen (Carlsbad, CA, USA). Primary antibodies against MEF2C and α‐actinin were obtained from Santa Cruz Biotechnology (Dallas, TX, USA), and primary antibodies against Nkx2.5 and troponin I were purchased from Abcam. Secondary antibodies were obtained from Bio‐Rad Laboratories (Hercules, CA, USA). All other chemicals were of analytical grade and used without further purification.

##### Synthesis of pDNA‐ and 5‐AZA‐Coupled CMC Nanogels

CMC was dissolved in filtered water. bPEI (MW = 25 000; Sigma) was dissolved in filtered water (50 mg per 10 mL). 5‐AZA was dissolved in filtered water (100 mg per 1 mL) and passed through a 0.2 µm filter. CMC (0.5 µg, 0.1% solution) was mixed with 5‐AZA (20 µM) and vortexed, and then the CMC/5‐AZA solution was mixed with bPEI (0.5 µg) at a 1:1 ratio and vortexed again. Cardiogenic vectors‐encoding GMT were complexed with CMC nanogels coupled to 5‐AZA. Each GMT vector also encoded EGFP. An NLS‐EGFP expression plasmid (pEGFP) was generated by ligating the EGFP open reading frame derived from pEGFP‐N3 into pcDNA3.1 (Invitrogen) followed by insertion of the SV40 NLS into the N‐terminus of green fluorescent protein (EGFP). All plasmid constructs were verified by DNA sequencing.

##### Characterization of CiCMC‐NPs

Sizes of CiCMC‐NPs were measured using a Zetasizer Nano ZS (Malvern, Southborough, MA, USA). Briefly, the nanogels were suspended in deionized water, and mean hydrodynamic diameter was determined by accumulation analysis. The zeta‐potential values were predicted based on the electrophoretic mobility of bPEI‐modified CMC nanogels in deionized water, which was evaluated using folded capillary cells in automatic mode. The viscosities of the various CiCMC nanogels were measured on a Brookfield Viscometer DV‐III Ultra (Brookfield Engineering, Middleboro, MA, USA) equipped with a programmable rheometer and circulating baths with a programmable controller (TC‐502P, Brookfield Engineering). The T‐F spindle was set to rotate at 0.2 rpm over a temperature range of 4, 25, 37, and 48 °C. To analysis of hydrogen ion by proton that was measured the hydrogen ion concentration range of pH 4, 5.5, 7.4, and 8.

##### Analysis of Cell Cycle Arrest by Flow Cytometry

NHDFs were seeded in six‐well plates at 1.5 × 10^6^ cells per well and cultured at 37 °C and CO_2_ (5%), after which they were rinsed twice and preincubated for 30 min with serum‐free DMEM‐high (2 mL) at 37 °C. CiCMC‐NPs were added to the cells and incubated for 4 h at 37 °C. After incubation, the NHDFs were washed twice with DPBS (1 mL) and treated with trypsin–EDTA (0.05%) for 5 min. The cells were resuspended in bovine serum albumin (BSA, 1 mL, 0.1%) and centrifuged at 13 000 rpm for 3 min. The cells were fixed for 1 h in ethanol (2 mL, 70%) on ice, after which they were centrifuged to removed supernatant. After three washes in DPBS, the cells were suspended in propidium iodide staining solution (1 mL, 10 µg mL^–1^, Molecular Probes) and RNase A (100 µg mL^–1^) in DPBS and incubated at 37 °C for 10 min. Samples were transferred to 96‐well plates, and fluorescence was measured by flow cytometry.

##### Evaluation of Transfection Efficiency with Cardiogenic Transcription Factors

NHDFs were seeded in six‐well plates (3 × 10^5^ cells per well) and cultured at 37 °C and CO_2_ (5%), after which they were rinsed twice and preincubated for 1 h with DMEM‐high medium (2 mL) at 37 °C. CiCMC‐NPs were added to the cells and incubated for 4 h at 37 °C. The NHDFs were then washed three times with PBS (1 mL) to remove any free gene complexes, suspended in PBS, and incubated for an additional 24 h. To determine transfection efficiency, the cells were harvested and analyzed on a flow cytometer (Guava Technologies, Hayward, CA, USA) equipped with a 488/642 nm laser. Data represent mean fluorescence signals from 5000 cells. For confocal microscopy, the cells were fixed with paraformaldehyde (4%). The cells were mounted in mounting medium (DakoCytomation, Hamburg, Germany) and visualized using a confocal laser scanning microscope (LSM 880 Meta; Zeiss, Germany). Fluorescence was monitored in the DAPI (excitation, 358 nm; emission, 461 nm), EGFP (excitation, 488 nm; emission, 530 nm), and CiCMC‐NP channels (excitation, 610 nm; emission, 655 nm). For Western blotting, the cells were rinsed twice in DPBS and resuspended in RIPA buffer (50 µL). Following electrophoresis on 10% (w/v) acrylamide SDS–PAGE gels, resolved proteins were transferred to membranes using a wet system. Membranes were incubated for 4 h at room temperature (RT) with primary antibodies diluted in blocking solution (cTnI, 1:500, Abcam; α‐actinin, 1:500, Santa Cruz Biotechnology), and then incubated for 1 h at RT with secondary antibodies. Binding was visualized using the Amersham ECL reagent (GE Healthcare, Pittsburgh, PA, USA), and signals were recorded on X‐ray film. Protein bands were quantified using the Image Lab software (version 4.0, Bio‐Rad) and normalized against β‐actin, used as a loading control. Total RNA was extracted using TRIzol (Thermo Fisher Scientific, Waltham, MA, USA). Reverse transcription was performed using synthesized cDNAs. Marker expression was confirmed by RT‐PCR using primers for GMT. RT‐PCR products were run on agarose gels (1.5%) and stained with ethidium bromide.

##### Immunofluorescence Analysis

iCMs were incubated for 4 h at RT with primary antibodies against cTnI (1:100, Abcam) and α‐actinin (1:500, Santa Cruz Biotechnology). Antibodies conjugated with Alexa Fluor 488 and Alexa Fluor 555 (Thermo Fisher Scientific) were used as secondary antibodies for cTnI or α‐actinin, respectively. Sections were mounted in mounting medium (DakoCytomation) and visualized on an LSM 880 confocal laser scanning microscope. Fluorescence was monitored in the Alexa Fluor 555 (excitation, 555 nm; emission, 565 nm), Alexa Fluor 488 (excitation, 495 nm; emission, 519 nm), and DAPI channels (excitation, 358 nm; emission, 461 nm).

##### Injection in Mouse Heart

The animal study was approved by the Institutional Animal Care and Use Committee (IACUC) of CHA (approval number: IACUC180104). After ICR mice (7‐week‐old, male; Orient) were prepared for surgery, the hair was removed, and the chest was cut to allow the heart to be pulled through the ribs. The prepared iCMs and nanogel were mixed 1:1 and injected through a syringe into the heart muscle, and the heart was collected 3 days later.

##### Evaluation of iCMs in Mouse Heart

Collected hearts were fixed for at least 1 day. The fixed organs were dehydrated and paraffin‐embedded. For observation of morphology, the samples were treated with Masson's trichrome (Sigma), Harris hematoxylin (Merck) and eosin, and nuclear stain.

##### Statistical Analysis

Data are representative results or the means of at least three independent experiments, ± SD. Statistical analyses were performed using two‐tailed Student's *t*‐test. Differences were considered significant at *p* ≤ 0.05, and *p*‐values are shown in figures as needed.

## Conflict of Interest

The authors declare no conflict of interest.

## Supporting information

Supporting Information

Supplemental Video 1
