# Supplementary material for: Direct Conversion of Human Dermal Fibroblasts into Cardiomyocyte‐Like Cells Using CiCMC Nanogels Coupled with Cardiac Transcription Factors and a Nucleoside Drug
Source: Adv Sci (Weinh). 2020 Feb 7;7(7):1901818. doi: 10.1002/advs.201901818 (PMC7141010; doi:10.1002/advs.201901818)
Supplement: Supplementary file 1 — Supporting Information [file ADVS-7-1901818-s001.pdf]

Copyright WILEY-VCH Verlag GmbH & Co. KGaA, 69469 Weinheim, Germany, 2020.

Supporting Information

for *Adv. Sci.,* DOI: 10.1002/advs.201901818

Direct Conversion of Human Dermal Fibroblasts into Cardiomyocyte-Like Cells Using CiCMC Nanogels Coupled with Cardiac Transcription Factors and a Nucleoside Drug

Hye Jin Kim, Hyun Jyung Oh, Ji Sun Park, Jung Sun Lee, Jae-Hwan Kim, and Keun-Hong Park**

Copyright WILEY-VCH Verlag GmbH & Co. KGaA, 69469 Weinheim, Germany, 2019.

Supporting Information

Direct conversion of human dermal fibroblasts into cardiomyocyte-like cells using CiCMC nanogels coupled with cardiac transcription factors and a nucleoside drug

Hye Jin Kim, Hyun Jyung Oh, Dr. Ji Sun Park, Jung Sun Lee, Prof. Jae-Hwan Kim, Prof. Keun-Hong Park

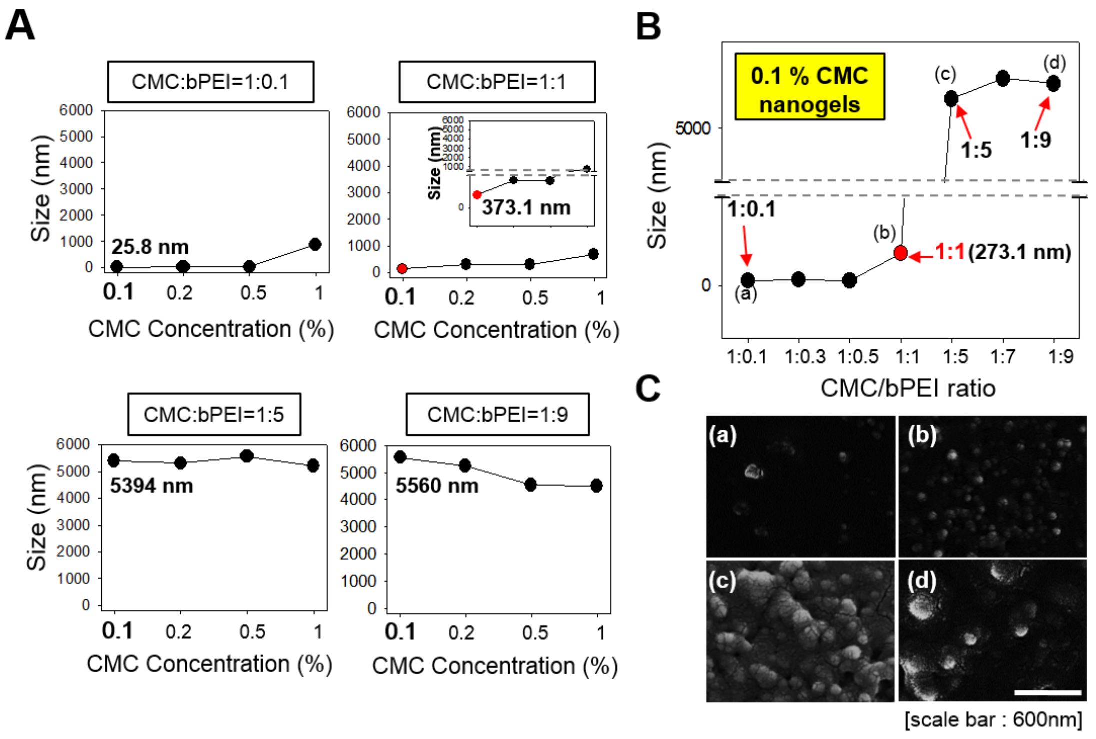

Figure S1. Size distribution and morphology of CiCMCs depends on the CMC:bPEI ratio. Size distribution of CiCMCs was determined by dynamic laser scattering (DLS) **(A and B)** and scanning electron microscopy (SEM) **(C)**.

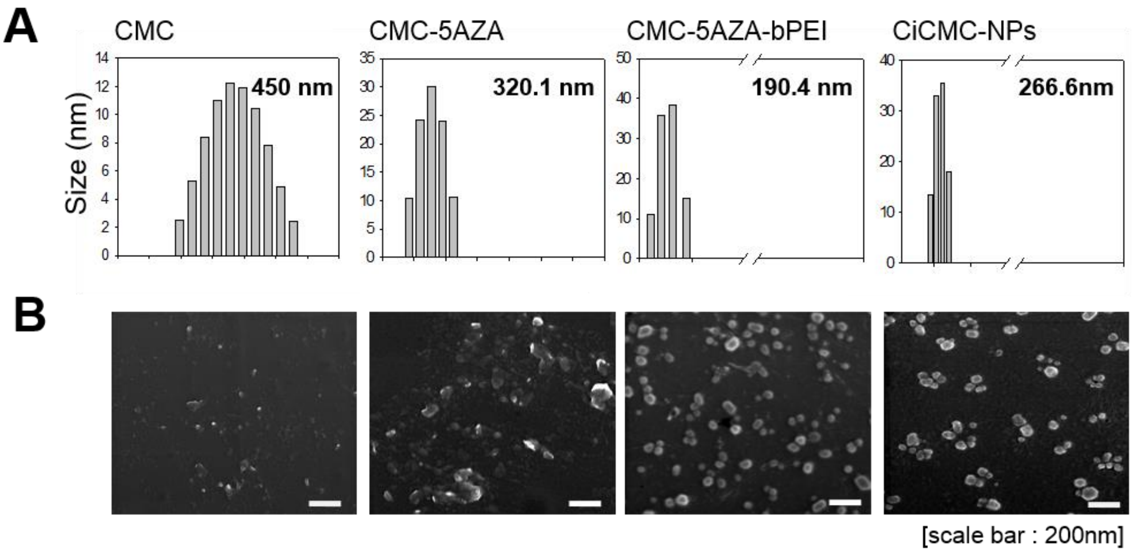

Figure S2. Size distributions of various types of nanoparticles and morphologies.
Size distribution of CMC, 5-AZA–loaded CMC, 5-AZA–loaded CMC complexed with bPEI, and CiCMC-NPs, as determined by dynamic laser scattering (DLS) **(A)** and scanning electron microscopy (SEM) **(B)**.

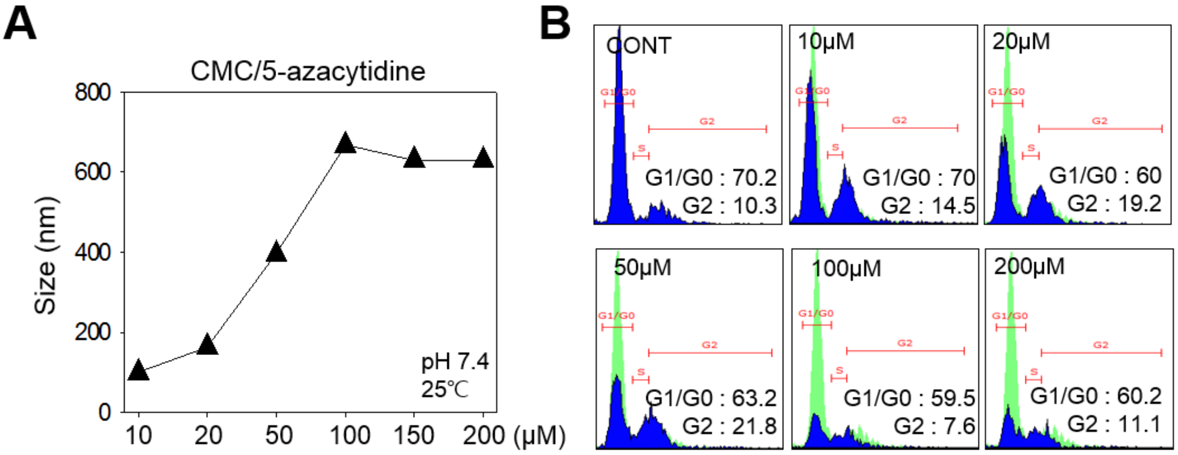

Figure S3. Size distribution of 5-AZA–loaded CMC-nanoparticles (A) and inhibition of proliferation in hMSCs (B).

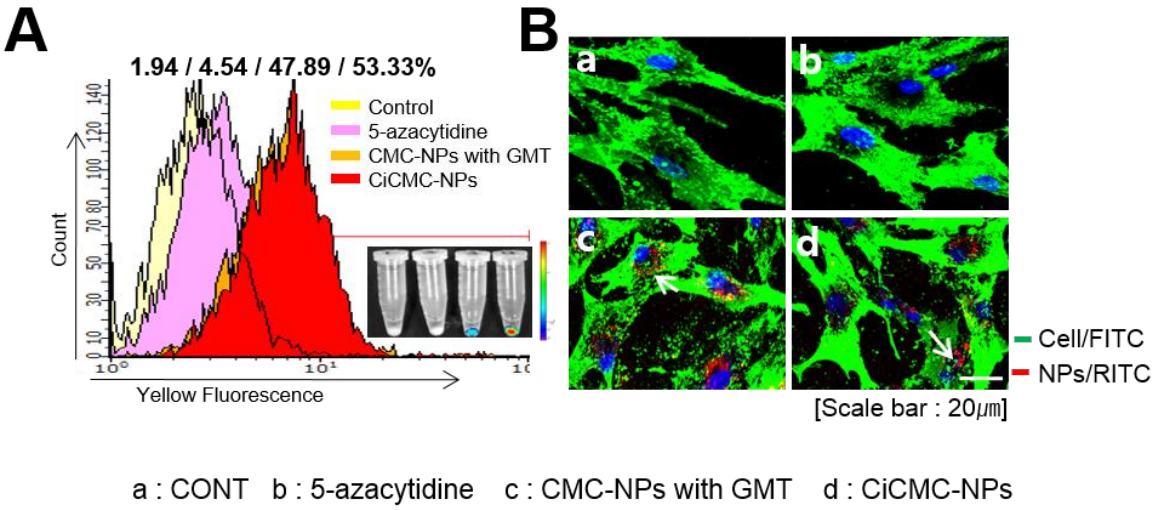

Figure S4. Efficiency of cellular uptake of several kinds of nanoparticles, as determined by FACS analysis (A) and confocal laser microscopy (B).

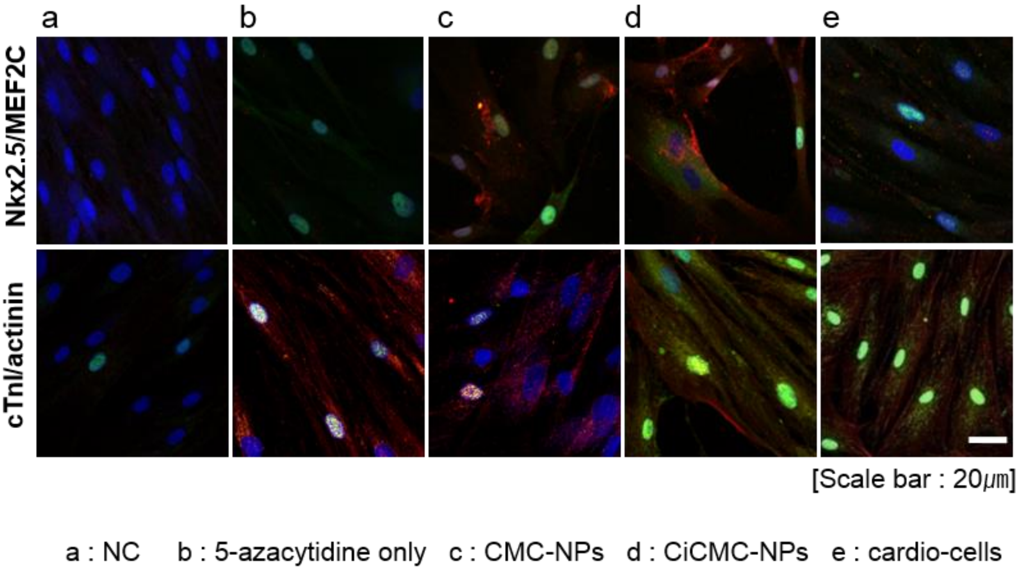

Figure S5. Expression of Nkx2.5, MEF2C, cTnI, and actinin in hMSCs following delivery of several kinds of nanoparticles.

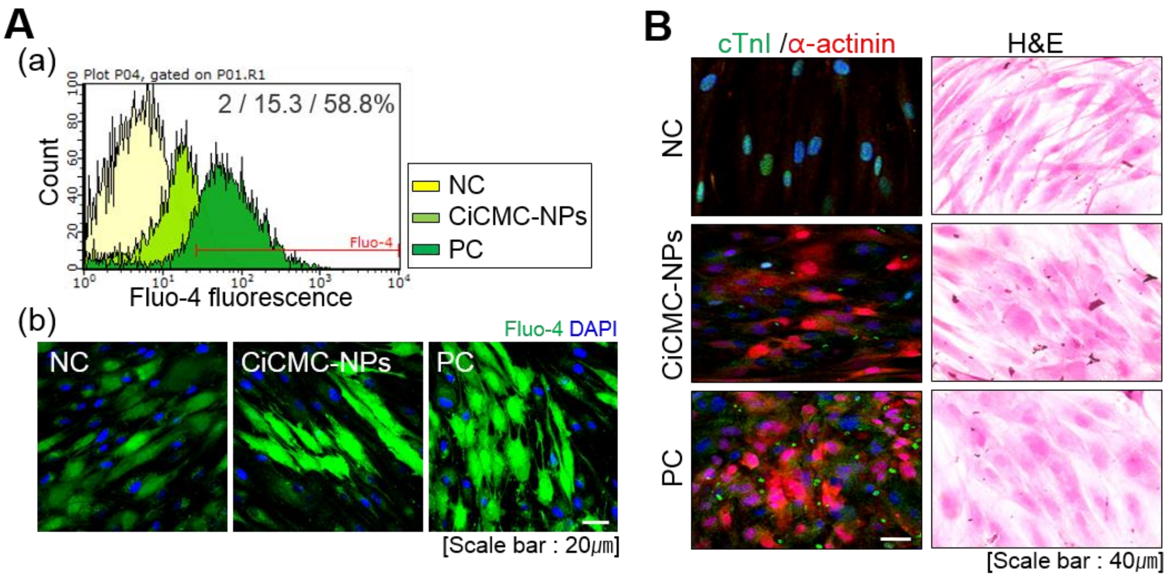

Figure S6. Evaluating the level of Fluo-4 fluorescence by FACS analysis (A, a), fluorescence imaging (A, b), and immunofluorescence imaging (B).
